# The complexity of polarization

**DOI:** 10.1073/pnas.2115019119

**Published:** 2022-04-21

**Authors:** Scott de Marchi

**Affiliations:** ^a^Department of Political Science, Duke University, Durham, NC 27708

Axelrod et al. (ADF; ref. 1) have a laudable goal: to create a simple model that reveals the causal mechanisms underlying polarization in order to better understand and “prevent extreme polarization.” All else equal, we should prefer simple models where comparative statics are straightforward. Policymakers desire monotonic effects, and models that produce highly conditional or nonlinear effects will get short shrift.

Models can, however, be too simple and fail to uniquely identify or test the causal mechanisms that would inform policy interventions. There are four reasons why the model presented by ADF (1) does not uniquely identify the mechanisms responsible for polarization.

**Fig. 1.**
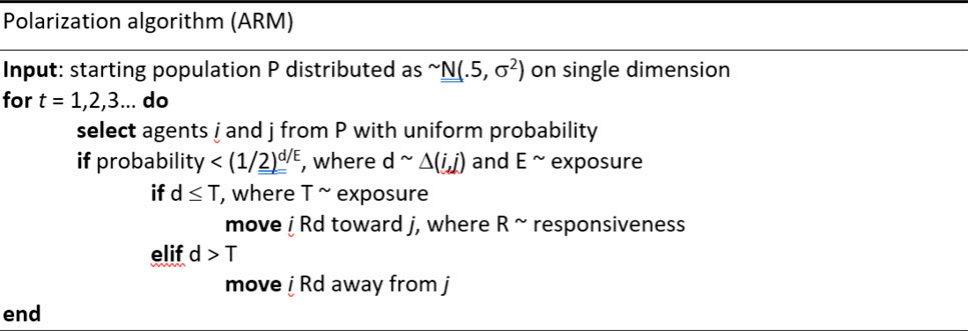
Polarization algorithm. ARM, Attraction–Repulsion Model. Reprinted with permission from ref. 1.

First, ADF believe the model depicted in Fig. 1 produces emergent/unexpected results. Yet, in this model the “distance” between the assumptions and results is quite small and behavior depends on global parameters. When tolerance is low the population splits into two modes at the edges of the ideological space. When tolerance is high, agents “stick” in the center. Similarly, low exposure or responsiveness slows the rate of change in the model. Is the finding that more tolerance leads to less polarization a case where local rules lead to “surprising” macrolevel results, or does this repeat conventional wisdom (2)?

Second, the model seems incomplete. ADF initialize their model with a centrist population but also state that the United States suffers from “growing animosities” akin to the Civil War. The logic of their model indicates that intolerance leads to a “runaway process” with “little hope of avoiding” extreme polarization. If their model is correct, what has kept the (currently centrist) US population from the expected polarized outcome? Is it that intolerance is less than some critical value? Or is the model missing dynamics promoting moderation?

Third, the best evidence from scholars working in this area indicates that the behavioral rules chosen by ADF are incorrect. Affective polarization, not policy polarization, is crucial (3). ADF argue that one could simply change the labels of the policy space to “parties” and keep the same dynamics—but is it plausible that the cognitive mechanisms underlying policy preferences and emotional attachment to parties (or political leaders) are identical? There is also substantial research on when interactions between opposed ideologies yield positive versus negative outcomes; none of this work indicates that such encounters are uniformly “repulsive,” as ADF assume (4).

Finally, it would be difficult to falsify ADF’s model. By selecting parameter values for T, E, and R, one can achieve end states ranging from polarization to centrism. Given the weight of the assumptions and lack of empirical tests, should we trust their recommendation that our democracy consider limiting citizens’ “exposure to dissimilar views”? Their conclusion is especially problematic given existing evidence that finds that limiting exposure increases rather than decreases polarization (5).

This raises more general questions. Can, as the authors argue, the simple algorithm in Fig. 1 explain such disparate phenomena as polarization, controversy over school desegregation, and the “rise of Hitler”? Or do overly simple models and the lack of hypothesis testing allow researchers to analogize too broadly (6)?
